# Surgical repair of large nasal septal perforation: a systematic review and meta-analysis

**DOI:** 10.1007/s00405-025-09913-9

**Published:** 2025-12-29

**Authors:** Matthew H. Cheung, Cory Hyun-su Kim, Shaun A. Nguyen, Michaela Close, Vusala Snyder, Michelle S. Hwang

**Affiliations:** 1 Department of Otolaryngology-Head and Neck Surgery, Medical University of South Carolina, Charleston, SC USA; 2 School of Medicine, SUNY Upstate Medical University, Syracuse, NY USA; 3 College of Osteopathic Medicine, Nova Southeastern University, Tampa Bay, FL USA

**Keywords:** Nasal septal perforation/Septal perforation, Surgical repair, Complications, Outcome, Success

## Abstract

**Purpose:**

Large nasal septal perforations (NSP) pose significant surgical challenges due to limited tissue availability, increased mucosal tension, and reduced vascularity. This systematic review and meta-analysis (SRMA) evaluates closure outcomes across surgical techniques for large NSP (≥ 20 mm) and answer in patients with large nasal septal perforations, how do different surgical approaches compare in terms of successful closure and failures?

**Methods:**

A SRMA were performed on studies employing surgical repair of large NSP (≥ 20 mm). Meta-analyses of continuous measures and proportions and comparison of weighted proportion were performed for demographic and outcomes.

**Results:**

Thirty-eight studies comprising 408 patients with large NSP were included. The mean perforation dimensions were 22.5 mm (length), 21.3 mm (width), and 24.4 mm (diameter). Common etiologies were iatrogenic (61.2%), idiopathic (36.5%), trauma (31.4%), and drug use (28.4%). Symptoms included nasal obstruction (62.0%), crusting (49.9%), and epistaxis (45.7%). Overall surgical closure rate was 84.4% (95% CI, 80.7–87.6). By technique, success rates were 88.2% for mucosal advancement, 86.6% for interpositional grafts, 72.7% for graft–flap combinations, and 91.8% for other approaches (pedicled/composite flaps, implants). Failures with mucosal advancement were predominantly reperforations, whereas interpositional and combination repairs more often failed due to inability to achieve initial closure.

**Conclusions:**

Surgical repair of large NSP achieves high success rates, though outcomes vary by technique. Mucosal advancement and interpositional grafts demonstrate comparable effectiveness, while graft–flap combinations perform less reliably. Larger defects remain the strongest predictor of failure, underscoring the importance of tailored surgical planning and careful technique selection.

## Introduction

NSP is a full-thickness anatomical defect in the nasal septum that spans 3 layers (mucoperichondrium, cartilage/bone, and mucoperichondrium), resulting in a communication between the two nasal cavities. NSP is a complex condition that can stem from various etiologies, including trauma, drug-related factors, chronic nasal infections, and autoimmune disorders. However, history of prior nasal septal surgery remains the most common underlying cause [1, 2]. The location and size of NSP are important factors determining whether patients remain asymptomatic or experience significant symptoms, such as nasal crusting, recurrent nosebleeds, and nasal obstruction [3–5]. While small perforations can often be managed conservatively, with options such as nasal saline irrigation and ointments, large perforations (≥ 20 mm in greatest dimension, as defined in surgical literature) pose a greater challenge for both patients and surgeons [5–7]. 

For patients, as perforations increase in size, laminar airflow is progressively disrupted, creating greater turbulence and causing increased subjective nasal obstruction [8–10]. For surgeons, the management of larger nasal septal perforations remains a complex endeavor, with a range of surgical techniques available, including flap advancement, cartilage grafting, and prosthetic implants [1, 6, 11]. While current literature provides insight into the outcomes of various surgical approaches in overall NSP management, a comprehensive review focused specifically on management of large septal perforations is lacking. This systematic review and meta-analysis aim to synthesize the available evidence on the surgical outcomes for the management of large nasal septal perforations.

## Methods

### Search criteria

This study was conducted in accordance with the Preferred Reporting Items for Systematic Reviews and Meta-Analyses (PRISMA) guidelines, and the review protocol was registered with the International Prospective Register of Systematic Reviews (PROSPERO, University of York, UK; registration numberCRD42024623499).

**Fig. 1:**
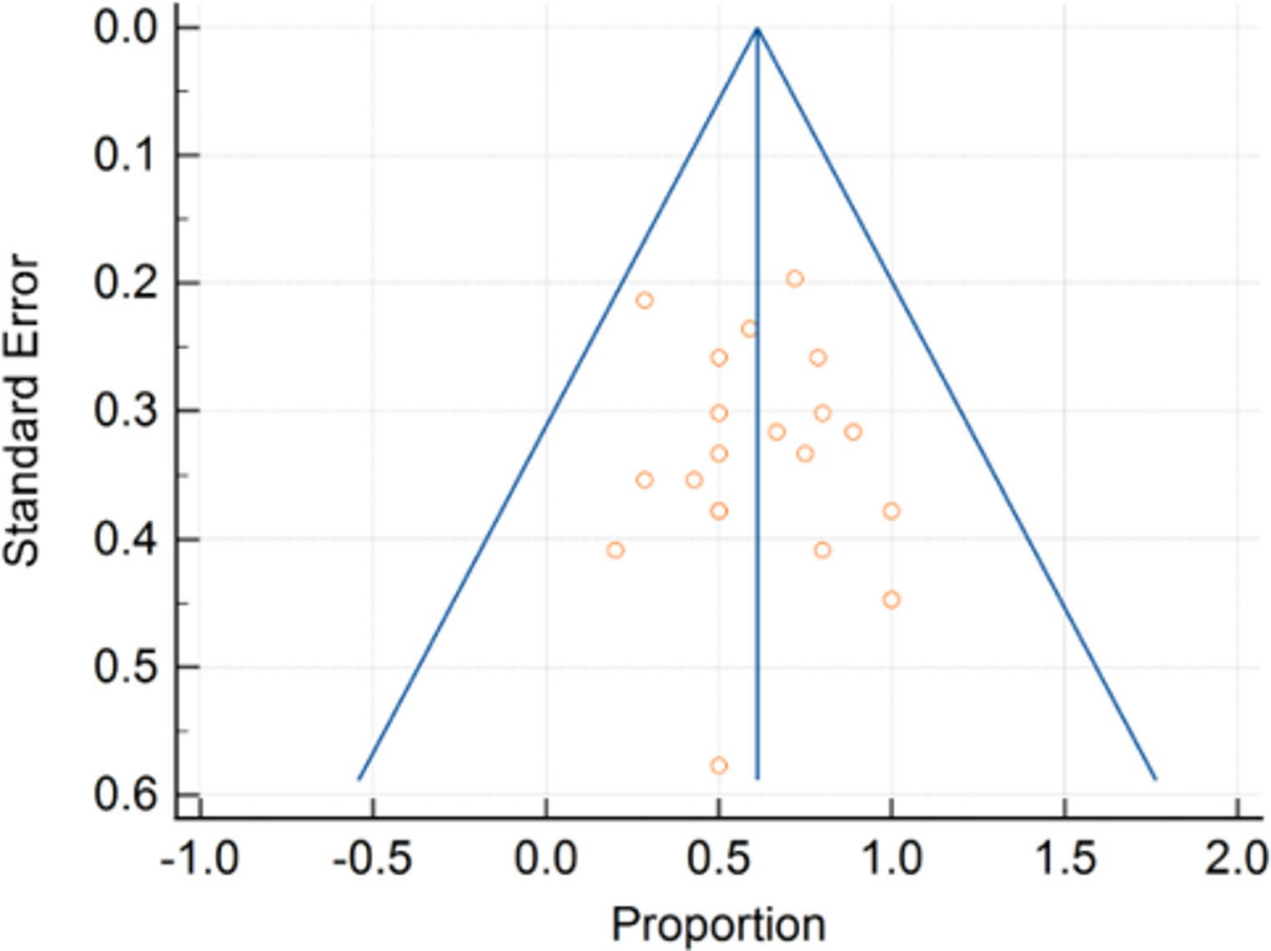
Funnel plot of proportions of male

Identification of studies for inclusion used keywords with detailed search strategies in databases including PubMed (U.S National Library of Medicine, National Institutes of Health), Scopus (Elsevier), and CINAHL. Databases were searched from their inception through 1/20/25. Search strategies used a combination of keywords for nasal septal perforation, septal perforation, mucosal flap, free flap, interpositional, septal button, septectomy, surgical, repair, complication, outcome, nasal septal perforation size and success. Pubmed search was then modified for other databases with appropriate subject headings and similar keywords. Detailed search strategies can be reviewed in Supplementary Data. The PRISMA diagram outlining the study review and inclusion process is presented in Fig. 1. References were uploaded to Covidence systematic review software (Veritas Health Innovation, Melbourne, Australia) for screening. Two reviewers, M.H.C and C.H.K, independently screened titles, and abstracts to determine eligibility. IRB approval was not required for this study as it was an analysis of publicly available, published data.

### Selection criteria

Studies were included in meta-analysis if they were written in English, assessed surgical outcomes of large nasal septal perforation, and/or reperforation incidence. Studies were excluded if no large septal perforation or size were reported. Studies includes retrospective cohort studies, case series and prospective cohort.

### Data extraction

Eligible full text was reviewed by the same reviewers for eligibility for this study. Data was independently extracted and compared for accuracy. Extracted variables included patient demographic and characteristics, study design, etiology, presenting symptoms, surgical technique and surgical outcomes. Perforation size was recorded according to reported measurements (anterior-posterior length, superior-inferior width, or overall diameter). When multiple dimensions were available, data for large perforations were extracted, and study-specific means were calculated. conflicts in data extraction were resolved by consensus between the two reviewers.

### Definitions

Large NSP defined as ≥ 20 mm in either AP, SI or overall diameter dimension. Successful closure defined as complete closure achieved at postoperative follow-up. Unsuccessful closure was defined as incomplete closure, failure to achieve initial closure or reperforation at any time during the follow-up period. Surgical techniques were categorized into four approaches: (1) Mucosal advancements defined as techniques involving repositioning of adjacent mucosa to cover the defect. (2) Interpositional graft defined as any techniques involving placement of graft material (e.g., cartilage, fascia dermis) between mucosal edges. (3) Graft-flap combination defined as techniques integration both mucosal advancement and graft placements. (4) Other approaches defined as techniques not otherwise classifiable (e.g. pedicled flaps, composite grafts and implants).

### Quality assessment

Articles were critically appraised to assess the level of evidence using Oxford center for Evidence-Based Medicine criteria. The risk of bias for each aspect was graded as low, high or unclear. Funnel plot for bias provided Fig. 1. For retrospective case series, the Joanna Briggs Institute (JBI) Critical Appraisal Checklist for Case Series was utilized [12] (Table 1). The components of the assessment were graded as “yes,” “no,” “unclear,” or “not applicable.” Components were given a score of “1” for “yes” and “0” for “no,” “un- clear,” or “not applicable.” The case series checklist was scored out of 10. Articles with scores of 5 or above were considered at low risk of bias and were, therefore, included in the systematic review and meta- analysis. Consistency of risk of bias assessment was check by two authors (M.H.C and C.H.K) and resolved by a third author (S.A.N).

**Table 1:** JBI Critical Appraisal Tool Case Series

	JBI Case Series										
Author	1	2	3	4	5	6	7	8	9	10	Overall
Alobid 2024	y	y	y	n	n	y	y	y	y	na	7
Altunay 2016	y	y	y	n	n	y	y	y	y	na	7
Ayshford 2003	y	y	y	n	n	y	y	y	y	na	7
Cavada 2019	y	y	y	n	n	y	y	y	y	y	8
Ceylan 2007	y	y	y	n	n	y	y	y	y	y	8
ÇomoğluŞ 2016	y	y	y	n	n	y	y	y	y	na	7
Emsen 2015	uk	y	y	uk	n	y	y	y	y	na	6
Epprecht 2017	y	y	y	y	y	y	y	y	y	na	9
Hancı 2024	y	y	y	n	n	y	y	y	y	na	7
Hanci 2021	y	y	y	n	n	y	y	y	y	y	8
Hanci 2024	y	y	y	n	y	y	y	y	y	na	8
Hanci 2020	y	y	y	n	n	y	y	y	y	na	7
Heller 2005	y	y	y	n	n	t	y	y	y	na	7
Hong 2016	uk	y	y	n	n	y	y	y	y	y	7
Hunter 2021	uk	y	y	n	n	y	y	y	y	y	7
Hwang 2022	uk	y	y	y	n	y	y	y	y	y	8
Kridel 1998	y	y	y	y	y	y	y	y	y	na	9
MoreraSerna 2017	N	y	y	n	n	y	y	y	y	na	6
Morse 2019	n	y	y	y	y	y	y	y	y	y	9
Ozdilek 2020	y	y	y	uk	n	y	y	y	y	y	8
Özer 2022	y	y	y	uk	n	y	y	y	y	y	8
Park 2013	y	y	y	uk	n	y	y	y	y	y	8
Pedroza 2007	y	y	y	y	y	y	y	y	y	y	10
Romo 1999	n	y	y	n	uk	y	y	y	y	y	7
Rusetsky 2024	n	y	y	n	n	y	y	y	y	y	7
Rusetsky 2020	y	y	y	n	y	y	y	y	y	y	9
Smith 2016	y	y	y	n	n	y	y	y	y	na	7
Taskin 2011	y	y	y	n	n	y	y	y	y	na	7
Taylor 2020	y	y	y	n	y	y	y	y	y	y	9
Woolford 2001	n	y	y	n	n	y	y	y	y	y	7
Zhang 2003	n	y	y	n	n	y	y	y	y	na	6
Zocchi 2022	y	y	y	n	n	y	y	y	y	y	8
1. Clear inclusion criteria? 2. Condition measured in standardized, reliable metrics? 3. Valid methods used for identifying condition? 4. Consecutive inclusion of participants? 5. Complete inclusion of participants? 6. Clear reporting of demographics? 7. Clear reporting of clinical information? 8. Outcomes or follow up clearly reported? 9. Clear reporting of presenting sites or clinical demographics? 10. Was statistical analysis appropriate?											

Y: yes, n: no, uk: unknown, na: not applicable

### Statistical analysis

Meta-analysis of continuous measures (age and NSP dimensions) and meta-analysis of proportions (patient demographics, etiologies, surgical approach, and complications) were performed by Comprehensive Meta-Analysis v4 (Biostat Inc. Englewood, NJ, USA). Each measure (mean/proportion (%) and 95% confidence interval [CI]) was weighted according to the number of patients affected. Random effects models were used due to presumed heterogeneity across the studies [13]. In addition, a comparison of means and proportions, expressed as difference (Δ) and 95% CI was done to compare outcomes between two groups. Finally, potential publication bias was evaluated by visual inspection of the funnel plot and Egger’s test, which statistically examines the asymmetry of the funnel plot [14, 15]. A *p* value of < 0.05 was considered to indicate a significant difference for all statistical tests.

## Results

### Study characteristics

**Fig. 2:**
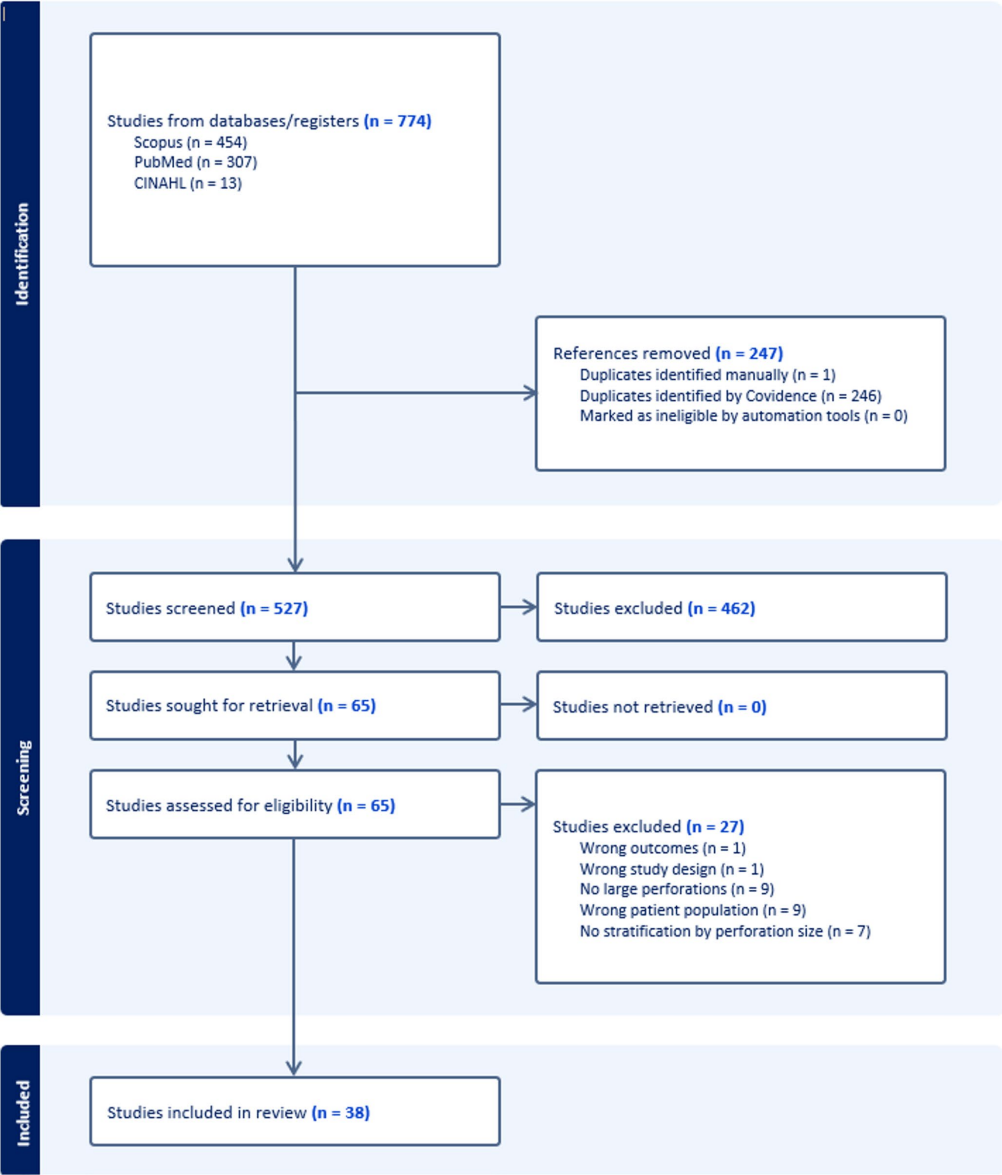
PRISMA

Electronic database search yielded a total of 774 articles. A total of 65 full text studies were assessed for eligibility in which 38 studies met the inclusion criteria. Studies were published between 1998 and 2024 (Table 1). The JBI (Supp Fig. 1.) critical appraisal indicated an overall acceptable low risk of bias for all case series (6 to 10 scores). A funnel plot with Egger’s test (1.23, 95% CI = -0.65 to 3.10, *p* = 0.19) demonstrated that all studies were within the funnel, suggesting little publication bias (Fig. 1). In the 38 eligible studies, a total of 919 patients were treated with surgical intervention for nasal septal perforation (Fig. 2). Studies included are provided in supplemental data. Of the 919 patients, 408 patients had large nasal septal perforations (NSP) and were included in the final analysis. Majority of the studies were retrospective case series. The mean age of the cohort was 38.6 years (range 15–73) with a mean follow-up period of 17.3 months (95%CI 15.9–18.8). 62.1% of the cohort were male. Study characteristics are shown in Table 2. Demographic data are shown in Table 3.

**Table 2:** Study characteristics

Author	Level of evidence	Large NSP patients (*n*)	Surgical technique	Approach	Approach	Follow up month (SD)	Success (%)
Alobid 2024	4	14	Boot on Donut	Endonasal	Mucosal Advancement	16 (8.5)	100
Altunay 2016	4	21	3D print prosthetic	n/a	Other	n/a	90.5
Ayshford 2003	4	6	Acellular human dermal allograft (alloderm) and an anteriorly based inferior turbinate flap	Endonasal	Combination	n/a	83.3
Cavada 2019	4	6	Anterior ethmoidal artery flap	Endonasal	Mucosal Advancement	3 (0)	100
Ceylan 2007	4	9	Mucoperichondrial flap	External	Mucosal Advancement	30.5 (19.9)	55.6
Chien 2024	3	28	n/a	n/a	n/a	n/a	78.6
Cho 2013	3	8	Polyethylene Implant & Mucoperichondrial Flap	Endonasal	Combination	12 (0)	87.5
ÇomoğluŞ 2016	4	6	Mucoperichondrium flap & Conchal Graft	Endonasal	Combination	n/a	50
Dayton 2017	3	7	Mucosoal Flap	Endonasal	Mucosal Advancement	n/a	100
Emsen 2015	4	10	Alar Wing Flap	External	Mucosal advancement	23 (12.6)	100
Epprecht 2017	4	7	Interposition Graft	Endonasal	Interposition Graft	11.3 (7.5)	71.4
Hadford 2023	4	5	Tri Layer Temporalis Fascia	External	Interposition Graft	4.2 (2.3)	100
Hancı 2024	4	25	Fascia Lata-fat Island Graft	External	Interposition Graft	12 (0)	92
Hanci 2021	4	13	Fascia Lata and Costal Cartilage Sandwich Graft	External	Interposition Graft	9 (0)	84.6
Hanci 2024	4	20	Fascia Lata Graft	External	Interposition Graft	n/a	90
Hanci 2020	4	5	Fascia Lata Graft	External	Interposition Graft	9 (0)	80
Heller 2005	4	6	Facial Artery Musculomucosal Flap	n/a	Other	17 (2.7)	100
Hong 2016	4	6	Advancement Flap + Rotation flap & Interpositional graft	External	Combination	46.8 (24.3)	66.6
Hunter 2021 (2–3 cm)	4	12	Rotation Flap and Interposition Graft	Endonasal	Combination	n/a	83.3
Hunter 2021 (> 3 cm)	4	4	Rotation Flap & Interposition Graft	Endonasal	Combination	n/a	0
Hwang 2022	4	14	Interpositional graft	External	Interposition Graft	36.6 (11.3)	85.7
Joo 2024	3	4	n/a	n/a	n/a		50
Kridel 1998	4	9	Interpositional graft	External	Interposition Graft	9.6 (2.8)	88.9
MoreraSerna 2017	4	4	Deep temporalis fascia graft sandwiched between two flaps	Endonasal	Combination	14.2 (2.5)	75
Morse 2019	4	3	Temporoparietal fascia graft & polydioxanone plate	External	Interposition Graft	2.9 (1.3)	100
Ozdilek 2020	4	4	Fascia Lata Graft	Endonasal	Interposition Graft	20.3 (11.1)	75
Özer 2022	4	22	Sandwich Graft	Endonasal	Interposition Graft		81.8
Park 2013	4	4	Advancement flap + Rotation flap & Interpositional graft	External	Combination	22.7 (16.6)	75
Pedroza 2007	4	21	n/a	n/a	n/a	n/a	90.5
Romo 1999	4	22	Mucosal Flap	External	Mucosal Advancement	n/a	81.8
Rusetsky 2024	4	22	Posterior Septal Artery Flap	Endonasal	Mucosal Advancement	22.3 (5.6)	81.8
Rusetsky 2020	4	6	Cross-septal Returned Flap	Endonasal	Mucosal Advancement	n/a	100
Smith 2016	4	5	Upper Lateral Cartilage Composite Flap - Pedicled flap	External	Other	n/a	100
Spatz 2024	3	10	Diced Cartilage in Fascia Graft	Both	Interposition Graft	n/a	100
Taskin 2011	4	17	Mucosal flaps & Auricular Interpositional grafts	n/a	Combination	15.2 (6.1)	94.1
Taylor 2020	4	2	Flap & Temporalis Fascia Interpostional Graft	External	Combination	10.5 (2.5)	100
Woolford 2001	4	8	Mucosal Flaps& Composite Cartilage Grafts	Both	Combination	21.3 (10.6)	87.5
Zhang 2003	4	5	Mucoperichondrial Flap	Endonasal	Mucosal Advancement	n/a	100
Zocchi 2022	4	8	Anterior Ethmoidal Artery Flap	Endonasal	Mucosal Advancement	n/a	62.5

n/a: not applicable

**Table 3:** Demographic & characteristics of population

	Proportion % (95%CI)
Male	62.1 (52.5–71.1)
Female	37.6 (28.6–47.1)
Age years (mean, SD)	36.7 (2.2)
Follow Up (mo)	17.3 (15.9–18.8)
Size mm (mean, SD)	
Overall	24.4(1.3)
Length (AP)	22.5 (6.0)
Width (SI)	21.3 (9.0)
Etiology	
Trauma	31.4 (22.0–42.0)
Drugs	28.4 (19.0-39.3)
Iatrogenic	61.2 (47.9–73.7)
Idiopathic/unknown	36.5 (27.0-46.9)
Symptoms	
Crusting	49.9 (30.0-69.7)
Nasal obstruction	62.0 (47.5–75.1)
Epistaxis	45.7 (35.0-56.7)
Whistling	25.2 (16.1–36.0)
Surgical approach	
Mucosal	25.1 (10.3–43.8)
Interpositional	34.0 (16.0-54.8)
Combination	25.0 (11.5–41.5)
Other	7.3 (2.2–15.1)

AP: Anterior-Posterior; SI: Superior-Inferior

**Table 4:** Closure rate by surgical approach

Approach	*n*	Closure % and 95CI
Overall	408	84.4 (80.7–87.6)
Mucosal	109	88.2 (78.1–95.4)
Interpositional	143	86.6 (80.9–91.5)
Combination	77	72.7 (47.7–85.4)
Other	32	91.8 (80.6–98.5)

### Size

16 studies measured size by length and width, 8 studies measured by diameter, 18 studies defined large NSP as ≥ 20 mm [6, 16–32]. Proportion analysis for average length, width and overall diameter were 22.5 mm (95%CI 21.5–23.5), 21.3 mm (95%CI 19.8–22.8) and 24.4 mm (95%CI 24.1–24.7) respectively. Data on large perforations for each study are shown in Table 2.

### Symptoms and complications

Meta-analysis identified four primary etiologies for large NSP: trauma, drug-use, iatrogenic and idiopathic cases. There were only 2 cases of vasculitis/autoimmune etiology, which were not categorized. The proportions for trauma, drug use, iatrogenic and idiopathic etiologies were 31.4% (95%CI 22.0–42.0), 28.4% (95%CI 19.0-39.3), 61.2% (95%CI 47.9–73.7) and 36.5% (95%CI 27.0-46.9). Proportions of commonly reported symptoms were 49.9% (95%CI 30.0-69.7) for crusting, 62.0% (95%CI 47.5–75.1) for nasal obstruction, 45.7% (95%CI 35.0-56.7) epistaxis, 25.2% (95%CI 16.1–36.0) for whistling. There were 6 reported cases of saddle nose deformity. Post-operatively, symptoms either all resolved or showed no complications in 87.2% (95%CI 79.1–93.3) of the cohort (Table 3).

### Overall surgical management

**Fig. 3:**
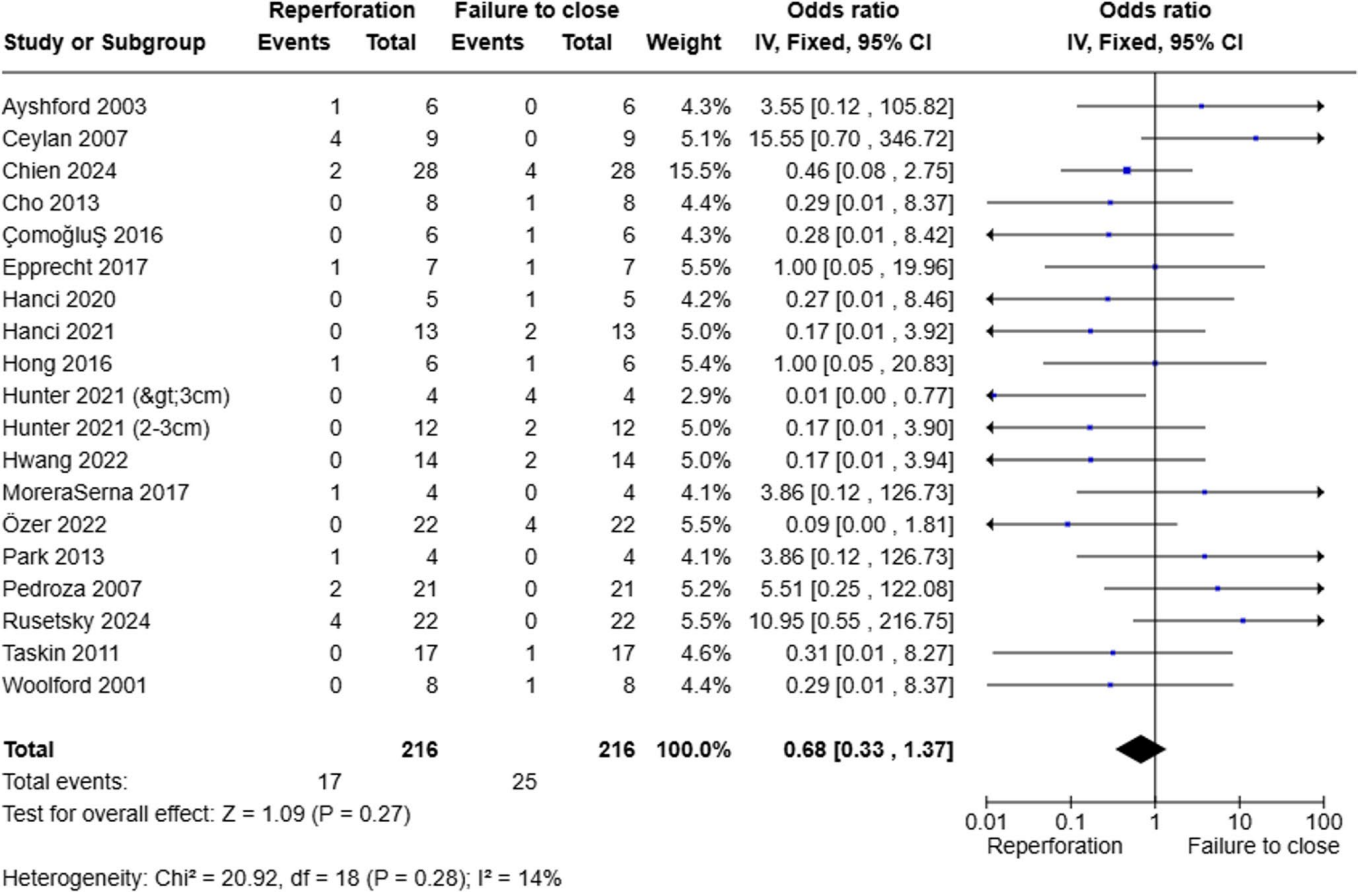
Odds ratio of failure events

Overall proportion for successful closure with surgical repair in large NSP was 84.4% (95%CI 80.7–87.6). Of the unsuccessful cases, reperforation was seen in of 40.5% (95%CI 22.0-60.4) and incomplete closure was seen in 61.4% (95%CI 42.1–79.0) within the reported follow-up period. All studies included outcomes for success/closure of perforations while 20 studies reported detailed data on unsuccessful outcomes with incomplete closure or reperforation. There was no significant difference in the odds of reperforation compared with initial failure to achieve closure (OR 0.68, 95%CI 0.33–1.41; *p* = 0.30). A forest plot comparing reperforation and failure to close is shown in Fig. 3. In our analysis, further stratification by surgical approaches identified three general approaches: mucosal advancements, interpositional grafts, and graft-flap combinations. Surgical techniques that did not fit into these categories were categorized into “other” which included pedicled flaps, composite grafts and implants. Allocation of studies into surgical approaches are shown in Tables 2 and 2 studies were excluded from stratification since no data on surgical technique were given or studies reported different techniques per patient. Of note. The total number of patients represented in each approach does not sum to the full cohort of 408. This is because stratification was based only on studies that explicitly reported the surgical technique used. Several studies did not specific the exact approach or reported multiple techniques without stratified outcomes and were therefore excluded from approach-specific subgroup analyses.

### Mucosal advancement

Mucosal advancement approach was utilized in 25.1% (95%CI 10.3–43.8) of cases, comprising 109 patients. The success rate was 88.2% (95%CI 78.1–95.4). The mean perforation dimensions in this technique were 24.5 mm in length (95%CI 24.1–24.9) and 23.8 mm in width (95%CI 20.2–27.4). Among unsuccessful cases 94.7% (95%CI 70.0-99.9) were due to reperforation, whereas only 5.3% (95%CI 0.1–30.0) represented failure to achieve initial closure.

### Interpostional graft

The interpositional graft approach was utilized in 34.0% of cases (95% CI, 16.0–54.8), comprising of 143 patients. The success rate was 86.6% (95%CI 80.9–91.5). The mean perforation dimensions were 20.3 mm in length (95%CI 18.5–22.1) and 19.6 mm in width (95%CI 17.6–21.6). Among unsuccessful cases, 81.3% (95%CI 47.0–95.2) represented failure to achieve initial closure, whereas 14.0% (95%CI 2.1–40.2) were due to reperforation.

### Combination

Graft–flap combination techniques were utilized in 25.0% of cases (95%CI 11.5–41.5), comprising of 77 patients. The success rate was 72.7% (95%CI 47.7–85.4). The mean perforation dimensions were 24.6 mm in length (95%CI 23.0–26.3) and 21.6 mm in width (95%CI 20.3–22.9). Among unsuccessful cases, 65.9% (95%CI 42.8–84.5) represented failure to achieve initial closure, whereas 34.2% (95%CI 15.5–57.2) were due to reperforation.

### Other

Three studies were categorized under other techniques, which were utilized in 7.3% of cases (95%CI 2.2–15.1), comprising of 32 patients. The success rate was 91.8% (95%CI 80.6–98.5). The mean perforation diameter for this category was 24.0 mm (95%CI 23.2–24.8).

**Fig. 4:**
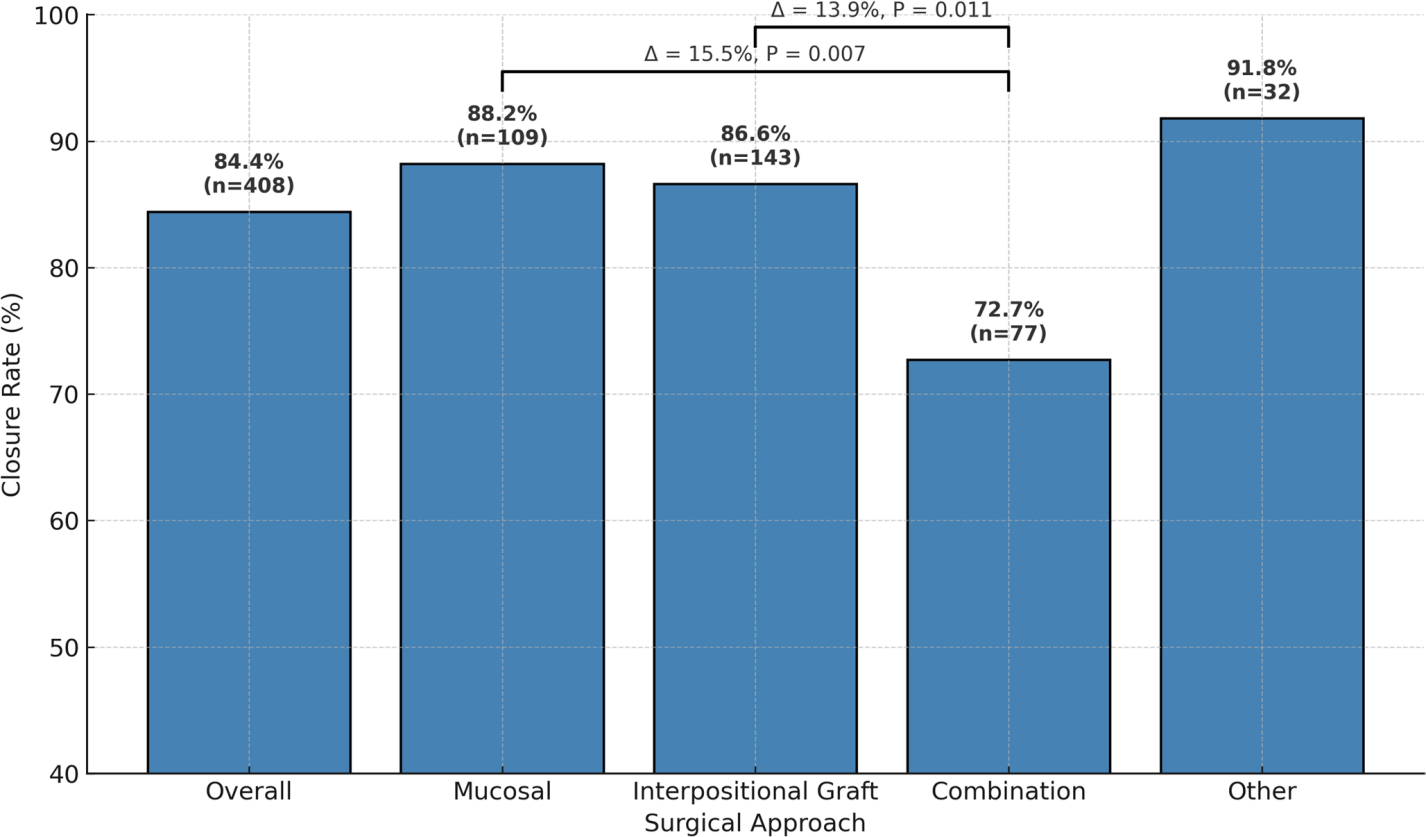
Closure rate and significance for large nasal septal perforation repair by surgical approach

When comparing approaches, there was no significant difference in closure success rates in interpostional graft and “other” approaches with mucosal advancement, which served as the reference group. However, mucosal advancement demonstrated a significantly higher success rate than graft-flap combinations with a mean difference of 15.5% (95%CI 4.08–27.4, *p* < 0.05). Similarly, interpostional grafts achieved significant higher success than graft-flap combinations, with a mean difference of 13.9% (95%CI 3.0-25.7, *p* < 0.05) (Fig. 4).

## Discussion

Large nasal septal perforation can be more technically challenging to repair compared to smaller perforations due to increased tension on closure and the larger distance needed for reperfusion. Although etiologies of large NSP-such as infections, trauma, chemicals, prior surgical history and autoimmune processes-remains similar to small perforation, large defects are complicated by limited tissue availability, increased mucosal tension, reduced blood supply, higher risk of persistent symptoms and the complexity of the required surgical techniques. Optimal surgical approach must facilitate tension-free closure, to maximize success and minimize reperforations [3, 33]. In this study we aimed to analyze closure rates in large NSP (≥ 20 mm) and compare outcomes across surgical approaches.

Our pooled analysis demonstrated an overall successful closure rate of 84.4%. This reflects the increased technical demands of large defect repair and is slightly higher than the 75% success rate reported in the 2012 SRMA by Kim et al. for large NSP, but lower than the 91% overall closure reported by Fermin et al. which included both large and small perforations [8, 34]. When stratified by perforation size, Fermin et al. reported closure of 90.1% in >1.5 cm defects and 92.2% in < 15 mm defects. While those results reflect broader landscape of NSP management, our focus specifically on larger perforations highlights the consistent trend that larger defects remain more difficult to close successfully.

Preoperative evaluation remains critical, including identification of etiology and symptom management. In our meta-analysis most common presenting symptoms in large NSP were crusting (49.9%), nasal obstruction (62.0%), epistaxis (45.7%) and whistling (25.2%). Symptomatic treatment alone may be the appropriate choice for some patients, or while awaiting surgical intervention [4]. Saline irrigation, nasal emollients septal buttons or hemostatic agents may provide temporary or adjunctive relief. More importantly, our review demonstrated that large NSP were most commonly iatrogenic in origin (61.2%), typically after prior septal surgery. Idiopathic or unknown etiologies comprised 36.5%, whereas autoimmune and infectious causes were rare. These finding suggest that surgical repair remains the definitive intervention for most patients with large symptomatic perforations [35]. Various techniques have developed over the past couple decades focusing on optimizing outcomes and addressing challenges that are associated with larger perforations.

### Mucosal advancements

Mucosal advancement techniques reposition surrounding mucosa to cover the defect [11, 36]. While traditionally suited to small or medium perforations, our analysis demonstrated an 88.2% success rate in large defects, highlighting its viability even in challenging cases. Failures were almost exclusively due to reperforation (94.7%), suggesting while initial closure was reliably achieved, long term stability of the repair remained the main challenge. Patients with healthier mucosa, limited prior surgical trauma and good vascularity may benefit most from this approach.

### Interpositional grafts

Interposition grafts provide structural support when mucosa alone is insufficient, with commonly used materials including cartilage, fascia or dermis [33, 37]. Our analysis found an 86.6% success rate. Failures were most often due to failure to achieve initial closure (81.3%), suggesting grafts may be particularly effective in preventing reperforation, although long-term outcomes depend on resistance to resorption or displacement [3, 33].

### Graft-flap combination

Combination techniques integrate both mucosal advancement and interpostional grafts to reinforce repair in larger or more complex perforations [3, 33]. Despite the theoretical advantage, our analysis showed a lower closure rate of 72.7%. Failures were more often due to failure to close (65.9%), likely reflecting a higher technical complexity of integrating two modalities, increased of flap tension and difficulty in establishing reliable graft vascularization. These finding suggests that although combination approaches may be reserved for the most challenging cases, they are also most vulnerable to initial technical failure.

### Other

Two studies using alternative techniques (e.g., pedicled and composite flaps, FAMM flaps, and implants) were grouped as ‘other,’ with a pooled success rate of 91.8%. Given the small sample size, these findings should be interpreted with caution.

Given these results, findings suggests that while mucosal advancement achieves the highest closures rates, both interpositional grafts and combination repairs remain valuable options depending on case complexity and patient factors. While interpostional grafts are avascular by nature, favorable outcomes are likely contingent on adequate vascular growth from healthy mucosal margins and careful patient selection. Conversely, graft-flap combination repairs, though theoretically advantageous, are frequently employed in larger or more complex defects where mucosal tension and scarring may predispose to initial closure failures. This likely explains the paradoxically lower success rates observed in our pooled analyses. Furthermore, studies reporting success rates approaching 100% likely reflect carefully selected cases, typically non-smokers with limited mucosal trauma and intact mucosa between the superior margin of the perforation and the nasal dorsum, thereby limiting their generalizability.

Across approaches mucosal advancement was associated with the highest success rate (88.2%) followed by interpostional graft (86.6%), and graft-flap combination at 72.7%. Mucosal advancement and interpostional grafts demonstrated comparable outcomes, whereas graft-flap combinations performed significantly worse, with a mean difference of 15.5% compared with mucosal advancement and 13.9% compared with interpostional grafts (both *p* < 0.05). Unsuccessful mucosal advancement cases were almost exclusively reperforations, whereas interpositional grafts and combination techniques more commonly failed due to inability to achieve initial closure.

These findings suggest that while mucosal advancement achieves the highest closure rates, repair integrity varies by approach, with interposition graft and graft-flap combinations more vulnerable to initial technical failures. The reduced performance of graft-flap combinations may reflect the increased technical complexity of integrating two repair modalities, higher risk of flap tension or challenges in vascularizing the graft component. However, in our pooled comparison of reperforation versus initial closure failure, there was no significant difference between the two outcomes (OR 0.68, 95% CI 0.33–1.41; *p* = 0.30). This suggests that both mechanisms of failure occur at comparable rates and reflect distinct challenges in the surgical repair of large perforations.

Of note, free tissue transfer and posterior septectomy are other viable approaches when treating large NSP with paucity of mucosa. Free tissue transfer introduces vascularized donor tissue, providing well-vascularized coverage, especially for large or complex perforations [38, 39]. However, when large posterior septal perforations cannot be repaired, posterior septectomy may offer a good alternative. Posterior septectomy, while not technically a repair, can alleviate symptoms by creating a single nasal airway and has demonstrated symptomatic improvement in up to 78% of patients [40, 41]. However, these approaches were not included in our study due to sparsity of data in free tissue transfer, and posterior septectomy not technically categorized as a perforation repair procedure.

While multiple approaches are available for repair of large NSP, approach selection not only impact closure success but also type of failures. Mucosal advancement and interpostional grafts provide more reliable outcomes, whereas graft-flap combination, despite their theoretical benefits appear to be more prone to initial failure. Larger perforation size remains the most consistent predictor of unsuccessful closure, as supported in prior SRMAs and reinforced in our analysis where we observed failure rate of 15.3%, improved from Kim et al. 2012 SRMA reporting 22.5% failure rate [4, 34]. 

It is also important to recognize that several key determinants of surgical success, such as perforation size and location, underlying etiology, and the quality and vascularity of the mucosa, were inconsistently reported across included studies. These factors play a major role in operative planning and long-term success of the closure; however, the lack of standardized and stratified data prevented formal statistical evaluation of their individual contributions to outcomes. Therefore, the results presented here should be interpreted within this context and not extrapolated to technique selection without consideration of patient-specific anatomic and etiologic factors.

### Limitations

We acknowledge the following limitations: There is variability among the included studies in defining and measuring large NSP. While some studies reported perforation size using anterior-posterior length and superior-inferior width, others used overall diameter or broad categorical classifications. While most studies reported specific perforation sizes, measurement methods were not consistently detailed. It was assumed that reported dimensions reflected direct measurement; however, variability in technique and reporting may introduce bias. This inconsistency may introduce bias in analyses despite our efforts to standardize data extraction. Therefore, we applied a random-effects model and conducted a meta-analysis of proportions. Additionally, the surgical techniques used for NSP repair varied across studies, therefore we categorized techniques into general approaches to improve consistency across studies. However, differences in individual study definitions may still contribute to variability in reported outcomes. To address this, we used a random-effects model in our meta-analysis to account for interstudy variability. Not all studies reported reperforation rates, affecting the comparability of success rates. To address this, we used a random-effects model and proportions in our meta-analysis to account for variability. Finally, this study relied on aggregate data from non-randomized studies rather than individual patient-level data. As a result, we could not perform subgroup analyses based on patient-specific factors such as comorbidities, prior treatments, or other risk factors that may influence surgical outcomes and surgical approach.

## Conclusion

In conclusion, surgical repair of large NSP remains technically challenging with an overall closure rate of 84.4%. Mucosal advancement and interpostional grafts achieved the highest and most comparable success rates, while graft-flap combination underperformed, reflecting their greater technical complexity and higher risk of initial failure to close. Larger perforation size continues to be the strongest predictor of unsuccessful repair, and therefore there is importance in selecting techniques that optimize tension-free closure.
